# SwarmDock and the Use of Normal Modes in Protein-Protein Docking

**DOI:** 10.3390/ijms11103623

**Published:** 2010-09-28

**Authors:** Iain H. Moal, Paul A. Bates

**Affiliations:** Biomolecular Modelling Laboratory, Cancer Research UK London Research Institute, Lincoln’s Inn Fields Laboratories, 44 Lincoln’s Inn Fields, London, WC2A 3LY, UK

**Keywords:** elastic network model, normal mode analysis, particle swarm optimisation, PSO, protein flexibility, RTB, CAPRI

## Abstract

Here is presented an investigation of the use of normal modes in protein-protein docking, both in theory and in practice. Upper limits of the ability of normal modes to capture the unbound to bound conformational change are calculated on a large test set, with particular focus on the binding interface, the subset of residues from which the binding energy is calculated. Further, the SwarmDock algorithm is presented, to demonstrate that the modelling of conformational change as a linear combination of normal modes is an effective method of modelling flexibility in protein-protein docking.

## 1. Introduction

Protein-Protein interactions are fundamental to almost all biological processes. Disruption of molecular recognition is integral to many diseases including cancer. Docking attempts to predict the structure of complexes from their monomeric constituents. The computational approach has the potential to confirm or dismiss putative interactions as well as provide structural knowledge which can be exploited for the design of therapeutic interventions in a range of diseases, should a sufficiently accurate model be produced.

The docking problem presents two main challenges; the generation of structures and the discrimination between structures with a scoring function. In this study, the focus is on the first of these challenges, for which a number of approaches have been used. The simplest method of docking two structures is to treat them as rigid bodies, usually using the Fast Fourier Transform (FFT) technique [1–8] which may include a refinement stage [9], although other rigid-body methods have been used [10,11]. For many proteins, however, there are flexible deformations upon binding which can alter their geometric and electrostatic properties. Attempts to cater for flexibility include rigid-body cross-docking of an ensemble of structures generated with molecule dynamics (MD) [12–14] and flexible refinement of rigidly docked poses [6,15–22]. Soft potentials have also been used to allow minor clashes [10,17,22], whilst others use a mean-field technique to select a conformations from a multiple copy representation [6,23] or assemble independently docked subunits connected by hinge regions [24]. A common flexible docking approach consists of an energy minimisation protocol (such as Monte Carlo, simulated annealing, simplex, MD, adopted basis Newton-Raphson or steepest decent) and a set of parameters to minimise (such as multiple copy weightings, atomic cartesian coordinates, internal coordinates or rotamers). In this paper, the degree to which low frequency normal modes can capture the unbound to bound transition is quantified, both on their own and in linear combination. A novel docking algorithm, SwarmDock, is explained and tested on a number of cases of varying difficulty.

Linear, harmonic vibrational motions around a single minima can be calculated using normal mode analysis. These motions may then be ordered by vibrational frequency. The lowest frequency eigenvectors correspond to the motions that can be excited with the least amount of energy and hence are the most accessible with thermal energy. The elastic network model simplifies the analysis, by only taking account of the shape of the molecule under analysis, an approximation which has shown to be capable of reproducing thermal B-factors at an atomistic [25] and coarse-grained [26] level of resolution, as well as NMR [27] and MD atomic fluctuations [28].

Despite the approximation only being valid for small linear motions and ignoring multiple optima and solvent dampening, many protein motions resemble a single low energy normal mode [29–32]. Dobbins *et al.* investigated normal mode analysis in the context of protein-protein docking. They showed the use of normal modes for predicting protein flexibility and demonstrated that global motions of high-flexibility proteins can, in many cases, be approximated with a single low energy normal mode [33]. While these studies have investigated the overlap between single normal modes and molecular motions, of interest in the context of constructing a docking algorithm is how closely a linear combination of normal modes can capture the conformational change upon binding, especially that of interface residues. Cui *et al.* used normal modes as a basis for understanding motion in the F_1_ ATPase molecular motor, using RMSD as a guide [34]. Petrone and Pande used a similar approach when analysing the motions of four proteins, including one protein-protein binding event [35], whilst Tama *et al.* have used elastic network normal modes to fit atomic structures to low resolution cryo-electron microscopy maps [36]. Mustard and Ritchie used a method related to normal mode analysis to study motions that occur upon the binding of 10 test complexes [37]. To date, there has been no investigation of the ability of normal modes to recreate binding associated conformational change at the binding interface, the subset of residues from which binding free energy is calculated.

Due to the reduced number of degrees of freedom, a linear combination of low frequency eigenvectors has been discussed as a method of modelling flexibility in docking algorithms [38–40]. Normal modes have been used to generate ensembles for cross-docking [41], relax docked structures [42,43] and dock small molecules [44–48]. Whilst these methods use normal modes in docking, the only algorithms to date that adjusts the coefficients of a linear combination of normals modes during the decent of the protein-protein binding funnel, are those that use the coarse grain model, ATTRACT [49] and FiberDock [50]. In the ATTRACT algorithm, up to 5 coefficients represent the amplitude along low frequency normal modes and the eigenvectors represent conformational degrees of freedom. A similar approach is used in FiberDock, in which 10 modes are selected based upon the repulsive forces acting between the binding partners.

Here, we investigate the ability of fine-grained, all-atom elastic network normal modes to analytically map various components of the unbound structures onto their equivalents in the bound structures using least squares linear regression and a large data set of 236 conformational changes upon binding [51]. As many protein interface refinement protocols do not optimise the backbone and sidechains concertedly, the ability of normal modes to capture conformational change of the backbone alone is also investigated. A normal mode representation of the backbone could be used in combination with a rotamer based side-chain optimisation algorithm, such as SCWRL [52]. Similarly, algorithms such as PULCHRA [53] can be used to reconstruct detail from a coarse-grained C*_α_* structural representation. Hence, we also perform the mapping at this level of resolution. Furthermore, we investigate the rotation-translation-of-blocks (RTB) method of calculating elastic network normal modes, and its ability to capture the unbound to bound transition.

During docking, concerted change in conformation, position and orientation are desirable. SwarmDock, a memetic docking algorithm in which the translational, orientational and conformational degrees of freedom are simultaneously optimised using the Particle Swarm Optimisation (PSO) metaheuristic [54], combined with a Solis and Wets local search algorithm [55], was developed to perform flexible docking using normal modes. The PSO algorithm is effective as a robust optimiser of non-linear, multi-modal functions. It has been successfully applied to many optimisation problems including neural network training, design optimisation, data mining [56], gene clustering [57] and sequence alignment [58]. More recently, PSO has been used for small molecule docking and has been shown to outperform methods based on simulated annealing and genetic algorithms [59–61].

In the SwarmDock protocol, the PSO algorithm is used to optimise coefficients of a linear combination of Hessian eignevectors, position and orientation. Local and global, flexible, unbound-unbound docking is performed on a number of complexes. Whilst SwarmDock has only been implemented with a simplistic energy function, it can successfully dock flexible structures which undergo significant conformational changes upon binding. The success of the algorithm as a function of the number of soft modes included for flexible deformation of the binding partners, is reported. A brief overview of SwarmDock has been given elsewhere [62], however the details of the algorithm are elaborated here for the first time.

## 2. Results and Discussion

### 2.1. Overlap

**Figure 1:**
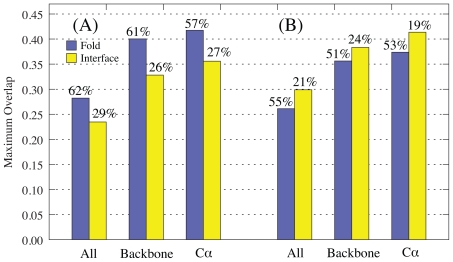
Mean Maximum overlap across the whole fold and the interface, for all atoms, backbone atoms and C*_α_* atoms, calculated with (A) standard diagonalisation of the Hessian and (B) the RTB approach. The percentage of complexes for which the mode of maximum overlap is one of the first 5 non-trivial modes is shown above the bars.

The intermolecular potential is determined by interacting residues, defined here as residues which have a non-hydrogen atom within 6 Å of a non-hydrogen atom on the binding partner. It is the ability of normal coordinates to capture the transition of these atoms that is most important for the purpose of docking. Figure 1A shows the mean maximum overlap (maximum overlap averaged over the test set) over the whole fold and at the interface, at three levels of resolution. As the size of the subsystem for which the overlap is calculated decreases, the mean maximum overlap increases. As expected, the difference between the backbone and the C*_α_* mean maximum overlaps is small, as backbone atoms move collectively with their adjacent atoms. The drop in mean maximum overlap when we compare backbone to all atoms demonstrates that whilst the side chains do move in concert with their adjacent backbone atoms, they can also move independently and change their rotameric state.

However, when we compare the mean maximum overlap of the whole protein to that of the interface, we observe a decrease at all levels of resolution. Furthermore, while the global motion is best represented by one of the 5 lowest frequency modes (57–62% of the modes of maximum overlap), conformational change at the binding interface is not (26–29% of the modes of maximum overlap). A Kolmogorov-Smirnov test shows that there is no evidence against uniformity in the distribution of modes of maximum overlap for the interface C*_α_* atoms (D = 0.070, D_0.01_ = 0.107). The same test for the global C*_α_* conformation shows that the mode of maximum overlap is not drawn from a uniform distribution above the 99% significance level (D = 0.368, D_0.01_ = 0.107).

**Figure 2:**
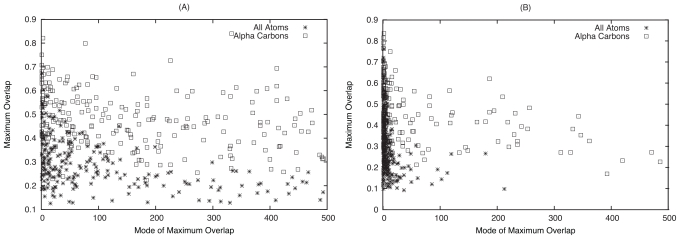
Maximum overlap and respective mode for (A) interface residues and (B) the whole fold.

**Figure 3:**
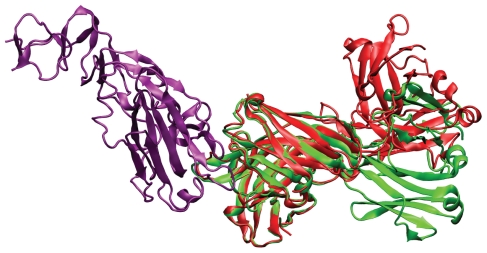
Complex 2VIS, of influenza hemagglutinin (purple) and murine IgG1, *λ* HC19 antibody in the bound (green) and unbound (red) crystal structures, superimposed on the binding domain.

This analysis was extended to the the modes of maximum overlap for the lowest 500 normal modes, and a difference was seen between the motion of interface C*_α_* atoms and all interface atoms (see Figure 2). However, when compared to the distribution of modes of maximum overlap for global motion, it is clear that much higher frequency modes are involved in the subtle changes that occur at the binding interface. This demonstrates that whilst the global change in protein conformation usually resembles one of the five lowest frequency normal modes, the conformational change of the binding interface does not. This is in agreement with Petrone and Pande [35], who suggest that higher energy modes can be activated by the presence of the binding partner. This discrepancy between the interface and global motion raises the question of the origin of the global change observed when comparing crystal structures. A dramatic example is given by the complex between influenza hemagglutinin and its paratope (complex 2VIS), as shown in Figure 3. The lowest frequency normal mode in the antigen binding protein corresponds to hinge bending motion which overlaps greatly with the unbound to bound transition. Whilst these types of conformational change can be involved in function, in this case the ligand binds away from the interdomain region and this motion does not correspond to the conformational change at the binding interface.

It has been shown that different crystal structures of the same protein can vary in their hinge bending angle, such as in the cubic and rhombic space group structures of the ligand free ribose binding protein [63]. Additionally, MD simulations of crystal structures of the T4 lysozyme, varying in their degree of hinge bending, have shown convergence of incipient structures after 500 ps [64]. Evidently, care must be taken when comparing crystal structures so as not to confuse binding associated change with crystal-packing induced conformational rearrangement facilitated by the intrinsic flexibility of the protein in question. Due to localisation of the binding interface, conformational change upon docking is expected to be less susceptible to this effect when considering the interface only, as this change is either not associated with global change, or the global movements are functional aspects of the binding, as in the case of a ligand binding to a cleft between two domains.

#### 2.1.1. Rotations-Translation-in-Blocks Method

The above analysis was repeated for modes calculated using RTB diagonalisation, and a comparison with modes derived from exact diagonalisation of the all-atom Hessian is shown in Figure 1B. The trend as we go to coarser levels of resolution in the fitting (all-atom to backbone to C*_α_*) is the same. However, there is a noticeable difference in the methods ability to model the conformational change at the interface compared to across the whole fold. The RTB method is better at capturing the local changes at the interface, with mean overlap values of 0.41 and 0.30 for C*_α_* and all-atom respectively. This exceeds the ability of the more computationally expensive standard Hessian diagonalisation method at capturing the interface unbound-bound change (0.36 and 0.23) and is on a par with its well known ability to capture global motions (0.42 and 0.28). The reasons for this are not clear, but may be related to the perturbation of low frequency modes with higher modes calculated at the residue level. Nevertheless, this does suggest that the RTB method is not only faster, but also a better choice for pre-calculating normal modes for use in docking.

### 2.2. Linear Combinations of Normal Modes

While overlap gives a good measure of how close a single normal mode is to the unbound-bound transition, it is not possible to know which mode has the greatest overlap without already knowing the bound structure. Hence, the inclusion of a number of modes is preferable for use in a docking algorithm. Furthermore, using these modes in linear combination may be able to significantly enhance the ability to recapture the important conformational changes that occur upon binding beyond that achievable by considering single modes, while still significantly reducing the search space compared to Cartesian or internal co-ordinates. In order to investigate the contribution of multiple modes, the unbound-bound transitions were decomposed into linear combinations of normal modes. It should be noted that, in terms of representing dynamics with a linear combination of normal modes, phase angles and amplitudes of motion must be considered. However, the purpose of flexible docking is not to model protein dynamics, but to model the conformational change that occurs upon binding. Hence, least square fitting is an appropriate method of combining modes in this context, and it is also probable that the coefficients generated by least-square fitting correspond to a structure accessible by a combination of harmonic motions.

**Figure 4:**
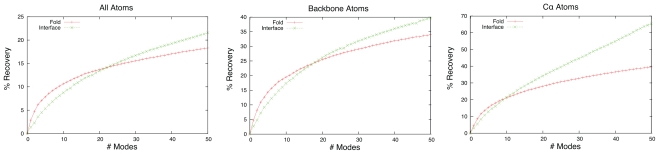
Mean percentage reduction in RMSD as a function of the number of modes used.

Linear least squares regression of unbound structures onto bound structures using a set of low frequency normal modes as a basis were performed. This yields the coefficients for the structure, of the infinite structures which can be generated with those normal modes as a basis, that minimises the RMSD against the bound form. Just as a certain subset of atoms, such as C*_α_* atom or interface atoms, can be used to determine the local overlap between a normal mode and the docking transition, subsets of atoms can be used in the fitting. Figure 4 shows the mean percentage reduction in RMSD, from the initial RMSD between bound and unbound, plotted against the number of low frequency modes used. Using 5 normal modes, only 44% of complexes have greater recovery in the interface than across the whole fold. This figure increases to 55%, 60% and 64% when using 10, 15 and 20 modes respectively. A similar trend can be seen when analysing the results for all atoms (41%, 45%, 50% and 52%). It is evident that as the number of modes used increases, the ability to model the conformational change at the interface improves at a greater rate than it does across the whole fold. This shows that although low-frequency modes have greater overlap with global motions than with local rearrangements at the interface, when taken in combination, conformational rearrangement at the interface can be better modelled than those across the fold, as more modes are included. This demonstrates that whilst a single mode of large overlap may well be of much higher frequency than those usually used to describe protein motion, greater recovery of RMSD can be garnered without the need to use hundreds of modes.

Generally, significant improvements in RMSD are observed in a subset of the modes considered, with other modes offering only a smaller improvement. For each protein, the mode which gives the greatest decrease in RMSD is the mode of greatest influence. Using twenty modes, the mean mode of greatest improvement for the whole fold is 6.8 and 5.0 when fitting the C*_α_* and all atom models respectively. These increase to 9.3 and 8.1 when only the interface is considered, in line with the overlap data shown in Figure 2 and further demonstrating the involvement of higher frequency motions occurring at the interface during binding.

**Figure 5:**
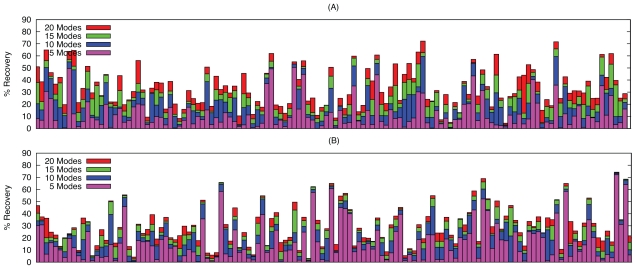
Percent reduction in RMSD upon analytical fitting of C*_α_* atoms to the unbound-bound transition, using 5, 10, 15 and 20 normal modes respectively as a basis. Values for the 130 proteins of highest initial C*_α_* RMSD, for both the interface (A) and the whole fold (B), are shown. The structures are ordered left to right in ascending order of initial RMSD.

Figure 5 shows the improvement in RMSD for C*_α_* interface and fold for a number of higher-flexibility proteins. A number of conclusions of can be drawn from this graph. When using 20 modes, the improvement in RMSD is generally greater for the interface than across the whole fold. Furthermore, the conformational rearrangements across the fold are usually modelled by the lowest 5 modes (purple), compared to the the higher frequency modes that are more often involved in conformational rearrangements at the interface (red and green).

**Figure 6:**
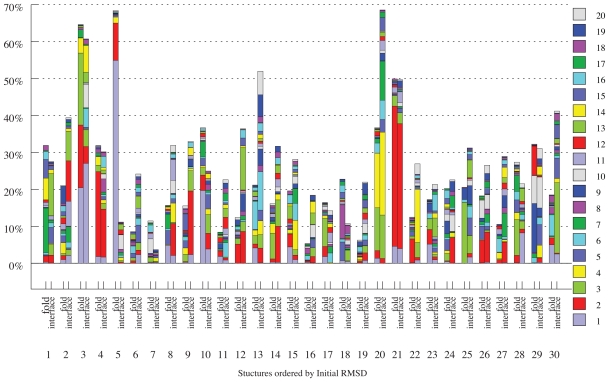
Percentage recovery of initial RMSD for 30 structures of greatest initial fold RMSD, ordered from left to right in descending order of initial RMSD, using all atoms in the fine-grained elastic network model. Effect of inclusion of 1 to 20 modes is shown for both the interface and the whole fold. Structures included are (1) 1IRA_r; (2) 1H1V_l; (3) 1Y64_r; (4) 1FAK_r; (5) 2VIS_r; (6) 1R8S_r; (7) 1IBR_r; (8) 1EER_r; (9) 1FC2_r; (10) 2FD6_l; (11) 2C0L_l; (12) 1FQ1_l; (13) 1JMO_r; (14) 1BKD_l; (15) 1GPW_r; (16) 1I2M_r; (17) 1YVB_l; (18) 2AJF_l; (19) 1NW9_r; (20) 1E4K_r; (21) 1IBR_l; (22) 2CFH_l; (23) 1HIA_l; (24) 2OT3_l; (25) 1KKL_r; (26) 1PXV_r; (27) 1KTZ_r; (28) 1BKD_r; (29) 1EER l and (30) 1IB1 r.

A more detailed breakdown of mode contributions for 30 complexes of high flexibility, as calculated using 20 modes, is shown in Figure 6. One of the most salient feature of this graph is structure 5, the influenza hemagglutinin and its paratope (structure 2VIS), discussed earlier. The global hinge-bending motion associated with the first mode is clearly unrelated to the conformational change occurring at the interface and most likely the influence of crystal packing forces. The distribution of modes for the interface is distinctly different from that of the fold for most cases, with some notable exceptions: I1BR (Ran/Importin *β* complex), 1E4K (immunoglobulin G Fc fragment/Fc*γ*RIII complex) and 1Y64 (actin/BNI1 complex), which all correspond to larger columns in Figure 6. For 1IBR, the receptor wraps around the ligand, which spans the entirety of the receptor. For 1Y64, the ligand bind to a cleft between two domains, which opens up with hinge bending motion. In 1E4K, the ligand binds to two distinct domains which again opens up with hinge bending motion. In these three cases, the global motion is functionally involved in the binding process.

It is interesting to note, however, that despite the differences between the global and local change, the predicted flexibility measure found by Dobbins *et al.* [33], which they showed to be effective at predicting global flexibility, can also be used to predict local flexibility. A Wilcoxon rank-sum test shows that, when ordered by C*_α_*, backbone and all-atom initial RMSD, the predicted flexibilities are not drawn from a different distribution, with significance of 0.0197, 0.0197 and 0.0006, respectively.

### 2.3. Docking as a Function of Normal Modes

In solution, the unbound proteins and the complex adopt an ensemble of conformational states. Much debate has focussed on the degree of overlap between the conformational ensembles of the binding partners unbound and in complex. At one end of the spectrum lies the ‘conformational sorting and population shift’ mechanism, in which members of the bound state ensemble are accessible by the unbound protein. At the other end lies the ‘induced fit’ hypothesis, in which bound conformations are essentially inaccessible by the unbound protein, but stabilised by the presence of the binding partner [65]. Significant evidence supporting the conformational sorting model come from a number of sources (see Boehr *et al.* and references therein). This gives credence to the idea of modelling flexible binding using information based upon the conformational diversity of the unbound binding partners, such as NMR ensembles, clustered MD snapshots, normal mode analysis or principle component analysis of MD trajectories. Such an approach is taken here. While induced structural changes undoubtedly play a role in molecular recognition, for many cases it is expected that the conformational diversity available using a set of pre-calculated low-frequency normal modes should be able to generate structures within or close to the bound ensemble, and with sufficient interaction energy to distinguish it from generated structures which do not correspond to the genuine biological interaction. Further, as higher frequency modes, which are visited less frequently in solution, are included, conformational change along these modes can be induced by the presence of the binding partner since, during the docking, both partners adjust their conformation in the presence of the field generated by the other.

**Figure 7:**
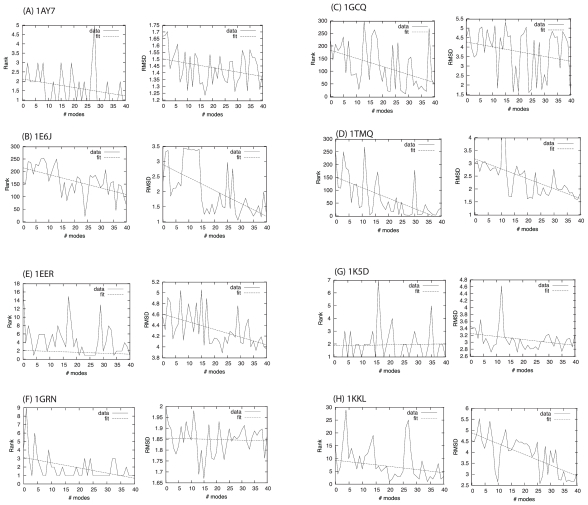
Docking results and linear regression for global docking (A–D) and local docking (E–H). I RMSD is the lowest value found in the docking run.

Global docking of four small complexes was done using up to 40 normal modes in both the receptor and the ligand, as calculated using the RTB method. For each complex, the SwarmDock algorithm was run 480 times at points spaced evenly around the receptor (4 times at each of 120 points surrounding the receptor). The rank and I RMSD are reported in Figure 7 (A–D). For the docking simulations involving a larger number of normal modes, structural distortions became apparent and so each structure was minimised in CHARMM prior to clustering to remove clashes and unphysical geometry in the internal structure. The general trend is shown by fitting a line to the data, all of which have a negative gradient for both the rank and the I RMSD, indicating that as more modes are included in the simulation, there is an improvement in the ability to discriminate correctly bound poses from false positives and capture conformational change at the interface. Interestingly, these complexes only undergo small backbone changes when comparing crystal structures. It may be the case that the inclusion of conformational dimensions preferentially broadens the correct binding funnel, enhancing the focus of the swarm, or deepening the energy minimum, as will be discussed shortly.

Local docking was performed for four high-flexibility models which undergo significant conformational change upon binding. In these docking runs, the search is focussed on the binding site region by performing more runs from the starting positions near the binding site and removing the starting positions away from the binding site. Of 120 positions evenly spaced around the receptor, the algorithm was run 8 times from the 10 positions nearest the binding site. For these complexes, 1EER, 1GRN, 1K5D and 1KKL, interface C*_α_* RMSD after superimposition of the interface, are 2.44 Å, 1.22 Å, 1.19 Å and 2.20 Å respectively. Rank and I RMSD are shown in Figure 7 (E–H). Low ranking conformations could be found for all these complexes, and the RMSD of the two most flexible complexes improved as more modes were included.

For many, but not all, of the false positives, the cluster size is small and they are not found consistently. However, the correct binding site is usually found multiple time during the docking runs. Consideration of the mechanism of the algorithm and the nature of biological interfaces offers a potential explanation. Structures near to the native complex also have low energies and, as a rule of thumb, poses further away from the binding site tend to have higher energies, resulting in an energy funnel surrounding the native complex. This is essential for the evolution of the interface, as it overcomes the binding equivalent of Levinthal’s paradox. SwarmDock is very different from other docking methods which rely upon finding low-energy structures by combining the results of many single independent trajectories through search space, or by filtering a list of putative structures using FFT correlations, geometric hashing or other scoring functions. Instead, communication between members of the swarm can result in, as an emergent property of the system, switching between exploration of diffuse regions of search space, or exploitation of narrower regions containing numerous positions which correspond to lower energy structures, depending on the nature of the energy landscape. SwarmDock takes advantage of a correlated energy landscape surrounding the true binding site, as low energy positions found by members of the swarm act as an attractor for a subset of the swarm. Further, the equation governing the velocities of the members of the swarm has a distance dependent repulsion term which acts against the contraction of a diffuse swarm, but has less effect on the contraction of a swarm which is focussing in on a particular region containing numerous low energy structures, such as the true biological interface. Indeed, previous work has shown that the mean Euclidean distance between the translational components of the particles (corresponding to the centres of mass of the ligands), decreases significantly earlier for SwarmDock runs which find the binding site compared to those that do not (data not published), indicating that fewer iterations are required to form a consensus in the population, and the presence of a wider binding funnel than for false positive sites - this information may be used in the future as part of a post-docking re-scoring step. It can now be seen that the inconsistent discovery of low energy false poses may not be because the number of starting positions is insufficient to find all low-energy poses, but because these poses are not surrounded by a wide binding funnel and hence not scrutinised by the swarm. Further information can be gleaned from the cluster size of the true positive cluster. Table 1 shows the average number of times the correct binding site was found during docking. For all but one of the complexes, the correct binding site was found most frequently in the runs with 21–30 modes or 31–40 modes included, despite the increasing dimensionality of search space. This is indicative that the width of the true binding funnel is increasing as higher modes are added, and is hence being found by SwarmDock more frequently during the search. This is consistent with the notion that the interface has evolved for efficient recognition of proteins, all of which posses greater or lesser degrees of intrinsic flexibility.

**Figure 8:**
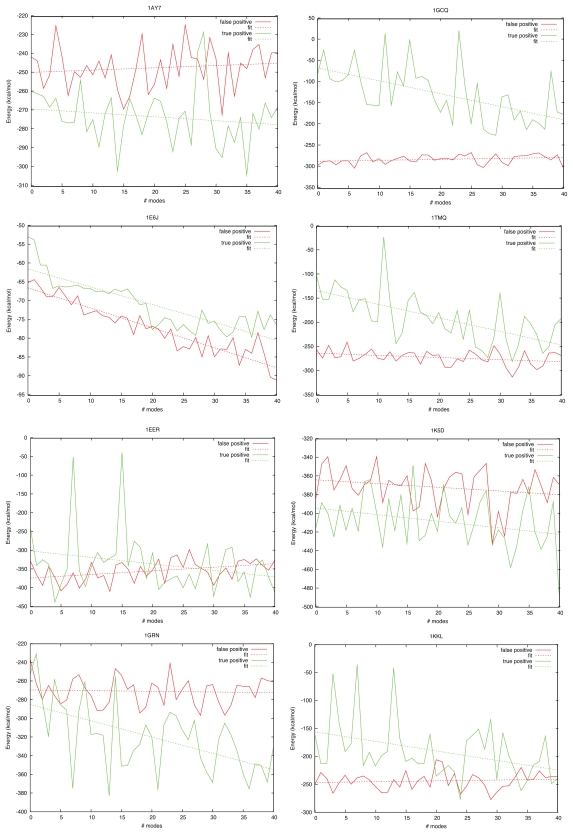
Energy of lowest energy true positive structure and mean energy of the lowest energy member of the 5 lowest energy false positive clusters.

Figure 8 shows how the energy of true and false positive clusters vary as more modes are included. It is interesting to note that, with the exception of 1E6J, the energy of the false positives did not decrease significantly as greater flexibility was added. However, the gradient of the fitted line for the true positive solutions was negative in all cases, and resulted in a significant lowering of interaction energy for most cases. This indicates that the true binding funnels are preferentially deepened compared to false positive binding funnels as higher modes are included.

As well as the examples given here, the SwarmDock algorithm has also been used in rounds 16–19 of the CAPRI blind docking trials. In the CAPRI experiment, the protein docking community test their algorithms blind. Target predictions are collected, the experimental bound structure revealed, and the predictions are ranked based on their quality. SwarmDock was able to successfully produce the bound structure of three target complexes: targets 37, 40 and 41. For these targets, semi-local docking was performed by eliminating certain regions around the receptor protein as potential binding sites, as described previously [62]. In target 37, acceptable predictions were made for Arf6 in complex with the LZ2 leucine zipper of JIP4 (pdb 2W83 [66]) by docking the unbound Arf6 (pdb 2A5D [67]) to a homology model of the leucine zipper built with the POPULUS server [68] using the GCN4 zipper as a template (pdb 2ZTA [69]). For target 40, both trypsin binding sites of the double-headed arrowhead inhibitor complex (pdb 3E8L) were predicted, one with medium accuracy and the other with high accuracy, by docking unbound bovine trypsin (pdb 1BTY [70]) to the bound form of the inhibitor. For target 41, a medium accuracy prediction was made for the Colicin E9/Im2 complex (pdb 1BXI), by docking the unbound Colicin E9 DNAse domain (pdb 1FSJ) to the unbound Im2 immunity protein (pdb 2NO8).

## 3. Conclusions

Conformational change upon complexation has been studied with the construction of docking algorithms in mind. The capability of fine-grained elastic network models to capture unbound to bound transitions was quantified by assessing the ability of normal modes to move all atoms, backbone (C, O, N, C*_α_*) and C*_α_* atoms from the unbound to bound conformation on a large test set. This analysis confirms the observation that the differences in the crystal structures of bound and unbound proteins can usually be approximated with a single low-frequency normal mode. However, both *in silico* and *in vivo*, protein-protein binding is driven by the lowering of the Gibbs free energy, which is dominated by the exclusion of water at the interface and the enthalpy of binding. Both these factors are largely determined by change that occurs at the protein-protein interface. Hence, the computational reconstruction of this change is of great importance when designing a docking algorithm. As previous studies have only looked at global motion, we paid close attention to the residues involved in binding. Global protein motion was shown to be distinct from localised conformational rearrangement at the interface, except in a minority of cases where global motion is functionally involved in docking. Despite this however, flexibility prediction designed to predict the extent of global change, can still be used to predict the degree of conformational rearrangement at the interface. Conformational changes at the interface correspond to motions associated with higher frequency modes than those associated with global structural rearrangements. While hundreds of modes need to be considered to find a single mode which sufficiently captures the unbound-bound transition at the interface, a linear combination of low-frequency normal modes is shown to do so with much fewer. Furthermore, despite involving higher frequency modes, greater improvement in RMSD can be made at the interface than across the whole fold, as increasing numbers of normal modes are used.

The effect of the RTB method was also investigated. While this technique is shown to be less accurate for predicting conformational change across the whole fold, it has a greater ability to model conformational change at the interface. For this reason, and due to its computational efficiency, this method makes a better choice for generating pre-calculated modes for docking. Fine-grained RTB normal modes were calculated and used in a novel docking algorithm, SwarmDock. SwarmDock is the first protein-protein docking algorithm based on particle swarm optimization, and was developed for flexible docking using normal modes as a method of modeling conformational change. The algorithm works well on the test cases presented here and in the blind CAPRI experiment. Further, RMSD and rank of docked structures can be demonstrably improved by the inclusion of normal modes, even for difficult cases and there is evidence to suggest that the true binding funnel is both widened and deepened, relative to false positive binding funnels, as higher modes are included. This demonstrates that the algorithm can effectively exploit the conformational freedom given by a set of low frequency modes. Further work, focussing on an improved scoring function and post-docking refinement and filtering, should further the ability for the SwarmDock protocol to generate potential bound structures and discriminate biological interfaces from artifacts.

## 4. Experimental Section

### 4.1. Normal Mode Analysis

In normal mode analysis, the potential energy basin is approximated as a harmonic well, allowing analytical solution to the equations of motion. Using a typical molecular mechanics energy function requires lengthy minimisation to ensure that the minima is found. To avoid this difficulty, we use the elastic network model (ENM) [25,26,32], with a Hookian potential used between close nodes, and the initial structure accepted as the minima.

$$U_{AB}=\frac{1}{2}k{\left(R_{AB}-R_{e,AB}\right)}^{2}\;\;\;U_{tot}=\sum_{R_{AB}<C}U_{AB}$$

Where the force constant, *k*, is equal for all atom pairs, *R_AB_* is the Euclidean distance between nodes *A* and *B* and *R_e,AB_* is their equilibrium separation. A cutoff, *C*, of 10 Å was used in all ENM calculations, where all atoms are nodes. The all-atom elastic network model is robust to cutoff [71], and the value used is large enough to dampen excessive motions of peripheral atoms, the tip effect. The Hessian matrix contains the second derivatives of the potential with respect to mass-weighted coordinates. Construction and diagonalisation of the Hessian matrix yields solutions in which all nodes have the same angular frequency, given by the square root of the eigenvalues. The respective motions are given by the normal coordinates, the eigenvectors. Any structural change can be constructed as a linear combination of normal coordinates, the complete set of which form an orthogonal basis. More detailed accounts are given elsewhere [72,73]. We use the package ElNeMo to construct the Hessian matrix [74] and diagonalise it using the Basic Linear Algebra Subprograms (BLAS) library [75]. All calculations were done on unbound structures, including all small molecule ligands, excluding crystallographic waters. Small molecules were removed from the results for the remainder of the analysis and for docking.

#### 4.1.1. The Rotation-Translation-of-Blocks Method

In the building block (RTB) technique for diagonalising the Hessian matrix, atoms are grouped into blocks, usually of one residue or more, which can translate and rotate as a rigid unit. The all-atom Hessian is projected into a block translation and rotation subspace by the use of a projection matrix. The projected Hessian is then diagonalised, yielding the vibrational frequencies and eigenvectors [76,77]. The eignevectors are then translated back into all-atom space by applying the transpose of the projection matrix, to produce approximate low-frequency modes. Subsequently, these approximate all-atom modes are perturbed toward the exact solution iteratively by accounting for higher frequency modes calculated for each block [78,79]. This method can reproduce frequencies and atomic fluctuations with accuracy comparable to that achieved when diagonalising the all-atom Hessian. Single residue blocks are used in this paper, as calculated using the diagrtb program distributed with ElNeMo [74].

#### 4.1.2. Overlap

The overlap value, *O_j_* describes how well the *j^th^* normal mode captures the displacement of atoms involved in a protein motion [80].

$$O_{j}=\frac{|\sum_{i=1}^{3n}a_{ij}(r_{i}^{b}-r_{i}^{u})|}{\sqrt{\sum_{i=1}^{3n}a_{ij}^{2}\sum_{i=1}^{3n}{\left(r_{i}^{b}-r_{i}^{u}\right)}^{2}}}$$

Where *r_i_^b^* and *r_i_^u^* are the *i^th^* coordinates of the bound and unbound structures respectively and *a_ij_* is the *i^th^* coordinate of the *j^th^* normal mode. Here, the maximum overlap, *O_max_*, is defined as the greatest overlap between an unbound to bound transition of the first 20 modes, unless stated otherwise.

#### 4.1.3. Data Set

The 124 complexes in the Protein-Protein docking benchmark v3.0 were used [51]. Both binding partners were analysed which, after removing the 12 complexes for which the unbound structure is only known for one of the binding partners, gives 236 conformation changes upon binding. All structures have a resolution below 3.25 Å with redundancy alleviated as described by Chen *et al.* [81].

### 4.2. Analytical Mapping of Unbound to Bound

This section describes the method used to determine the extent of deformation along each normal mode in order to generate the closest structure to the bound state from the unbound state. When fitting unbound structures to the bound structures using normal coordinates as a basis, a one-to-one correspondence of atoms is necessary. For some complexes, the bound and unbound structures have this correspondence. For the remainder, sequences of the two structures were aligned and non-matching residues were ignored in the mapping, but not in the construction of the Hessian matrix. When considering subsets of the structure, such as interface or backbone only, all atoms not in that subset are also ignored, leaving the *n* atoms of interest. When investigating the ability of an orthogonal set of vectors to capture protein motion, the contribution of each mode can be calculated independently as a projection [37,43]. However, when investigating atoms which do not correspond to the complete set from which the modes were calculated, such as the interface, orthogonality can no longer be assumed and an alternative approach in necessary. Hence, the unbound structures were mapped onto the bound structures using linear least squares regression, *ie* the unbound-bound transition is decomposed through basis expansion of a limited number of modes. The transition, **T**, from unbound structure, **D**, to bound structure **E**, is fitted to a linear combination of normal modes, *f*(*i, β*) = ∑*_j_*_=1_*^m^* *β_j_***M***_ij_*, by finding the minimum sum of square residuals, *S*.

$$T=E-D=r+\sum_{j=1}^{m}\beta_{j}M_{j}\;\;\;S=\sum_{i=1}^{3n}r_{i}^{2}$$

As the sum squared residuals are quadratic with respect to the coefficients, its derivative with respect to the coefficients is linear, with one unique solution which, as the square residuals are convex, correspond to a minimum.

Setting the gradient to zero,

$$\frac{\partial S}{\partial \beta_{j}}=2\sum_{i=1}^{3n}r_{i}\frac{\partial r_{i}}{\partial \beta_{i}}=0\;\;\;\text{for }\;\;j=1\ldots m$$

which can be arranged to a series of *j* normal equations,

$$\sum_{i=1}^{3n}\sum_{k=1}^{m}M_{ij}M_{ik}\beta_{k}=\sum_{i=1}^{3n}M_{ij}T_{i}\;\;\;\text{for }\;\;j=1\ldots m$$

Written in matrix form and inversion shows how the analytically fit coefficients, *β*, are obtained. These coefficients correspond to the magnitudes of deformation along their respective normal coordinates.

$$(M^{T}M)\beta =M^{T}T\;\;\;\beta ={\left(M^{T}M\right)}^{-1}M^{T}T$$

**Figure 9:**
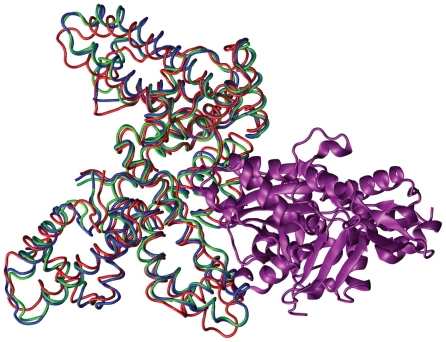
The complex, 1KXP, between actin (purple) and vitamin D binding protein (VDBP) in the bound (green) and unbound (red) conformations. A fitted structure (blue), obtained by linear regression to the VDBP unbound to bound transition, using 20 normal modes as a basis, has a C*_α_* RMSD of 0.87 Å compared to an initial RMSD of 2.12 Å. This corresponds to the closest structure to the bound that is attainable using the 20 lowest non-trivial normal modes.

Fitting was done for all 136 unbound-bound transitions in the data set, an example from which is shown in Figure 9.

### 4.3. SwarmDock

**Figure 10:**
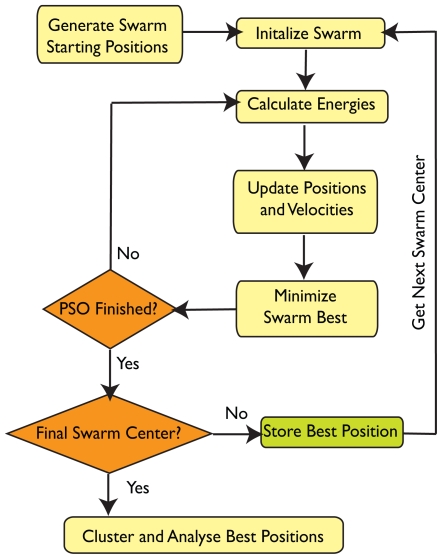
An overview of the SwarmDock algorithm.

The SwarmDock algorithm is a novel iterative population based mimetic algorithm. Each member of the population, *i*, is a particle in search space with both position in search space, ***ξ****_i_*, and velocity, **v***_i_*. Upon each iteration, the energy of each particle is evaluated and the positions and velocities are updated. Additionally, the lowest energy particle undergoes a local search based on that of Solis andWets [55]. A typical SwarmDock run takes around 10 minutes on a 2.66 GHz CPU. A schematic of the algorithm is given in Figure 10.

#### 4.3.1. Search Space

The search space is composed of the ligand centre of mass (3 translational dimensions) and a quaternion representation of ligand orientation (4 orientational dimensions). The receptor is kept fixed at the origin. *N_r_* and *N_l_* normal mode coefficients (*N_r_* + *N_l_* conformational dimensions) are also included for the receptor and ligand respectively. Hence, ***ξ****_i_* is a vector pointing to a position in 7 + *N_r_* + *N_l_* dimensional search space.

#### 4.3.2. Initialisation

SwarmDock is run at approximately evenly spaced positions surrounding the receptor, generated using a point distribution algorithm explained elsewhere [62]. The translational component of each particles position is moved randomly away from this position (drawn from a Gaussian *σ* = 10 Å) and each particle is assigned a random orientation. Normal mode coefficients for the initial swarm are drawn from a Gaussian. To ensure reasonable internal bonded geometry of the initial swarm, normal mode coefficients are drawn from a low distribution (*σ* =3.0) and the algorithm searches from here. Throughout this paper, the same number of modes are used in the receptor and the ligand.

#### 4.3.3. Propagation

The energy function is evaluated for all members of the initial population, then the velocities and positions are updated using the following transition functions, a variation on the well studied PSO equations [54]. These are used iteratively to propagate the algorithm.

$$\begin{array}{c} \chi_{i}(t+1)=\chi_{i}(t)+v_{i}(t+1)\\ v_{i}(t+1)=wv_{i}(t)+c_{1}r_{1,i}(p_{i}(t)-\chi_{i}(t))+c_{2}r_{2,i}(p_{n,i}(t)-\chi_{i}(t))+r_{3,i}(p_{\text{rand}}(t)-\chi_{i}(t)) \end{array}$$

where *w*, the inertial weight, is the degree to which previous velocity contributes to the new velocity. *r*_1_*_,i_*, *r*_2_*_,i_* and *r*_3_*_,i_* are random numbers taken from a uniform distribution between 0 and 1. Parameters *c*_1_ and *c*_2_ are respectively known as the cognitive and social aspect, both set to 2.05. The cognitive aspect is the propensity for the particle to move toward the lowest energy position it has previously experienced. The social aspect is the degree to which the particle moves toward the lowest energy position found by any particle in the neighbourhood of the particle being updated. **p***_i_* and **p***_n,i_* are position vectors that correspond to the lowest energy position found by particle *i* and the best position found by any particle within the neighbourhood of particle *i*, respectively. **p**_rand_ is the position of a randomly selected particle.

Note that the use of the word neighbourhood has a specific, non-colloquial meaning, as it appears in the PSO literature. A particle is deemed to be in the neighbourhood of particle *i* if it is one of the *k* particles adjacent to particle *i* in the vector array of the computer memory, where *k* is the neighbourhood size, which is set to 114 for all docking runs, out of the total population of 350. Neighbourhoods wrap around in the standard way, so that particles connected to their neighbours form a ring network topology.

#### 4.3.4. Velocity Clamping

To avoid explosions and control the exploration/exploitation trade-off, velocity clamping is used to impose a limit on the distance the particles can move in any one iteration:

$$v_{ij}(t+1)=\{\begin{array}{ll} v_{ij}^{\prime } & \text{if}-V_{j,max}<v_{ij}^{\prime }<V_{j,max}\\ V_{j,max} & \text{if }v_{ij}^{\prime }>V_{j,max}\\ -V_{j,max} & \text{if }v_{ij}^{\prime }<-V_{j,max} \end{array}$$

where *v′_ij_* is the calculated velocity of iteration *t* + 1. *V_max_* is set at 5 Å for translational parameters, 0.2 rad for the angular term in the quaternion and 0.5 Å for spatial terms in the quaternion. As the conformational parameters do not reach excessive velocities, no clamping is set on the normal mode coefficients.

#### 4.3.5. Local Minimisation

At each iteration, the lowest energy particle in the swarm undergoes a local search step which is based on that of Solis and Wets [55]. This is an adaptive minimisation technique which does not require gradient information. Its adaptivity arises from its ability to adjust its step size according to the number of previous successes or failures. Additionally, there is a bias vector in search space, **b** which gives preferential searching in the direction that has previously been most successful.

The algorithm works as follows. For each dimension in search space, *j*, a deviate is taken from a gaussian distribution centered around the bias *b_j_*, with a standard deviation *ρ_j_* . These deviates form a vector **d**. The energy of the particle is evaluated at ***χ*** + **d**. If this energy is lower than the energy at position ***χ*** then the bias vector is adjusted according to the equation below, the success counter is incremented and the fail counter is set to zero. If not, the energy is evaluated at ***χ*** − **d** and if lower in energy than at ***χ*** then again, the bias is updated, the success counter is incremented and fail counter is set to zero. If a lower energy position is not found then the success counter is set to zero and the fail counter is incremented.

$$b(t+1)=\{\begin{array}{ll} 0.6b(t)+0.4d & \text{if }E(\chi +d)<E(\chi )\\ 0.6b(t)-0.4d & \text{if }E(\chi +d)>E(\chi )\;\text{and }E(\chi -d)<E(\chi )\\ b(t)/2 & \text{if }E(\chi +d)>E(\chi )\;\text{and }E(\chi -d)>E(\chi ) \end{array}$$

If the success or fail counters reach five, then the the step size is expanded or contracted, by doubling or halving the standard deviations, ***ρ***. Initial values for *ρ_j_* are 0.5 Å for the translational parameters, 5° for the angular term in the quaternion, 0.25 Å for the spatial terms in the quaternion and 0.15 for the normal mode coefficients. The algorithm is repeated and terminates after 5 consecutive contractions.

#### 4.3.6. Clustering

After a set number of iterations, the lowest energy complex found is returned. The results for all runs are clustered. The lowest energy structure forms the first cluster. In ascending order of energy, all subsequent structures are added to a cluster if they are within 2.5 Å RMSD of the first structure in any existing cluster, otherwise they form a new cluster. A ranked list of structures is returned, with each structure corresponding to the lowest energy member of the cluster.

#### 4.3.7. Analysis of Docked poses

The analysis of the quality of docked poses uses interface RMSD, a metric that is used in the community-wide CAPRI experiment [82]. Two residues are deemed to be in contact if they have a pair of atoms within 5 Å of each other. Interface residues are those in contact with the binding partner. The interface RMSD, I RMSD, is defined as the minimum RMSD between the backbone atoms of the predicted structure and the crystal structure, just considering the interface residues.

#### 4.3.8. Energy Function

A simple energy function is employed in the docking algorithm, composed of a van der Waals term and a Coulombic term between *i* and *j* interacting atoms, on the receptor and ligand respectively. A switching function is used between 7 and 9 Å (*r_on_* = 7 and *r_off_* = 9), in order to eliminate discontinuity in the energy function and avoid calculating long-distance interactions with negligible contribution to the interaction energy.

$$\begin{array}{c} E_{int}=\sum_{i}^{atoms}\sum_{j}^{atoms}E_{i,j}\\ E_{i,j}=\{\begin{array}{ll} \frac{q_{i}q_{j}}{ɛr_{i,j}}+\sqrt{{ɛ}_{i}{ɛ}_{j}}\left[{\left(\frac{R_{min_{i,j}}}{r_{i,j}}\right)}^{12}-{\left(\frac{R_{min_{i,j}}}{r_{i,j}}\right)}^{6}\right] & \text{if }r_{i,j}<7\\ \left(\frac{{\left(r_{off}-r_{ij}\right)}^{2}(r_{off}+2r_{i,j}-3r_{on})}{{\left(r_{off}-r_{on}\right)}^{3}}\right)\left[\frac{q_{i}q_{j}}{ɛr_{i,j}}+\sqrt{{ɛ}_{i}{ɛ}_{j}}\left[{\left(\frac{R_{min_{i,j}}}{r_{i,j}}\right)}^{12}-{\left(\frac{R_{min_{i,j}}}{r_{i,j}}\right)}^{6}\right]\right] & \text{if }7<r_{i,j}<9\\ 0 & \text{if }9<r_{i,j} \end{array} \end{array}$$

All parameters are taken from the Charmm19 force field [83]. No potential was used to model the internal energy. While structures very rarely wandered into regions of search space which correspond to significantly distorted structures, this did result in some structural deformations when higher modes were included in the calculation. However, the structures were extensively minimised (500 steps conjugate gradient, 4000 steps adopted basis Newton-Raphson and 600 steps steepest decent) in CHARMM prior to final interaction energy calculation, clustering and analysis, ameliorating perturbations in structural integrity. In the future, changes in internal geometry may be penalised during the simulation in accordance to the extent of deformation along each mode and the eigenvalue association with that mode, so that a more diverse initial swarm can be generated.

## Figures and Tables

**Table 1:** The mean cluster size for the correct binding site (I RMSD *<* 5 Å), averaged over runs including different numbers of modes. The greatest values are marked in bold.

Complex	Modes: 1–10	11–20	21–30	31–40
1AY7	**15.1**	11.2	12.3	14.3
1GCQ	1.2	1.9	**2.9**	2.6
1E6J	10.0	9.9	11.0	**11.5**
1TMQ	2.1	2.9	**4.7**	2.9
1EER	3.6	3.1	5.3	**5.9**
1K5D	5.9	6.9	**7.4**	6.6
1GRN	26.6	28.7	**29.3**	27.3
1KKL	2.8	2.9	3.9	**5.6**
